# Why Are Functional Amyloids Non-Toxic in Humans?

**DOI:** 10.3390/biom7040071

**Published:** 2017-09-22

**Authors:** Matthew P. Jackson, Eric W. Hewitt

**Affiliations:** School of Molecular and Cellular Biology and Astbury Centre for Structural Molecular Biology, Faculty of Biological Sciences, University of Leeds, Leeds LS2 9JT, UK; m.p.jackson@leeds.ac.uk

**Keywords:** amyloids, fibril, oligomers, toxicity, functional amyloid

## Abstract

Amyloids were first identified in association with amyloidoses, human diseases in which proteins and peptides misfold into amyloid fibrils. Subsequent studies have identified an array of functional amyloid fibrils that perform physiological roles in humans. Given the potential for the production of toxic species in amyloid assembly reactions, it is remarkable that cells can produce these functional amyloids without suffering any obvious ill effect. Although the precise mechanisms are unclear, there are a number of ways in which amyloid toxicity may be prevented. These include regulating the level of the amyloidogenic peptides and proteins, minimising the production of prefibrillar oligomers in amyloid assembly reactions, sequestrating amyloids within membrane bound organelles, controlling amyloid assembly by other molecules, and disassembling the fibrils under physiological conditions. Crucially, a better understanding of how toxicity is avoided in the production of functional amyloids may provide insights into the prevention of amyloid toxicity in amyloidoses.

## 1. Introduction

Amyloid fibrils are cross-β assemblies and were first identified in amyloidoses, an array of devastating human diseases [1,2,3]. To date, over 30 different sequences are now known to misfold to form amyloids in amyloidoses, including amyloid-β (Aβ) in Alzheimer’s disease, α-synuclein in Parkinson’s disease, huntingtin in Huntington’s disease, and β_2_-microglobulin (β_2_m) in dialysis-related amyloidosis (DRA) [4,5,6,7]. Crucially, amyloid formation can result in degeneration of the affected tissue [5,8]. This intimate association with human disease led to amyloid fibril assembly being considered as a pathological process. It was therefore surprising to discover that amyloid fibrils perform physiological functions in organisms ranging from prokaryotes to humans [9,10]. In humans, functional amyloids have been proposed to participate in an array of physiological processes including pigmentation, the storage of peptide hormones, the fertilisation of oocytes by sperm, antimicrobial responses, regulated necrosis, and cellular responses to stress [11,12,13,14,15,16,17,18,19,20,21]. Nonetheless, the toxicity of amyloid formation in amyloidoses raises an important question that this review article will address, namely, how under normal circumstances can human cells produce functional amyloid fibrils without suffering any obvious deleterious effects?

## 2. Amyloid Fibril Assembly and Structure

**Figure 1:**
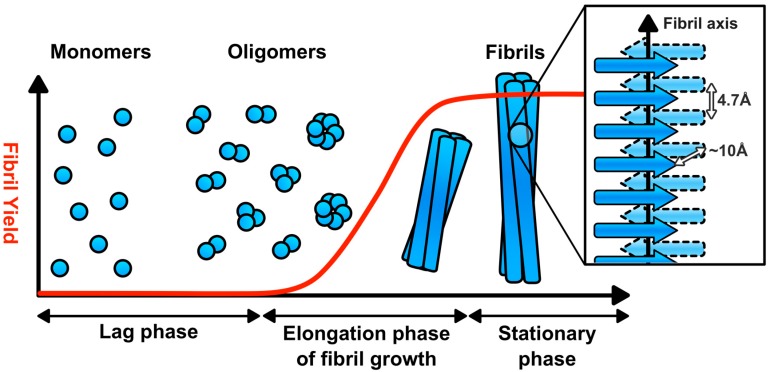
Amyloid fibril assembly. Amyloid fibril assembly is nucleation dependent. There is an initial lag phase, populated by prefibrillar oligomers, which ends with the production of the fibril nucleus. Fibrils then assemble in an exponential manner until the amyloidogenic precursor is exhausted. The resultant fibrils are composed of two or more protofilaments. Within each protofilament, the subunits have a cross-β core, corresponding to β-sheets aligned perpendicular to the long-axis of the fibril.

The assembly of amyloid fibrils from their peptide and protein precursors is a nucleation dependent process [6,7]. There is an initial lag phase, which ends in the formation of a fibril nucleus (Figure 1) [6,7]. This initiates the exponential phase in which fibrils assemble, before fibril growth then plateaus in the equilibrium phase [6,7]. The resultant amyloid fibrils are unbranched fibres of 5–15 nm in width, composed of two or more protofilaments and can be many microns in length [6,7]. Within the protofilaments, the individual subunits form a cross-β structure, corresponding to ribbon-like arrays of β-sheets that are aligned perpendicular to the long axis of the fibril (Figure 1) [6,7,22]. The cross-β structure of amyloid fibrils produces a distinctive X-ray fiber diffraction pattern, with reflections at 4.7 Å and ~10 Å, corresponding to the hydrogen bonding distances between β-strands and side chain packing between the sheets, respectively [3]. Amyloid fibrils also have characteristic tinctorial properties, binding to the dyes thioflavin-S, thioflavin-T, and Congo red, the latter exhibiting green birefringence in polarised light when bound to amyloids [3]. In addition, antibodies that recognise conformational epitopes in amyloid fibrils and their assembly intermediates have been generated [23,24,25].

Although the core of all amyloid fibrils is a cross-β structure, and hence the fibrils share some common properties, the precise arrangement of the subunits in the protofilaments is dependent on the precursor [6,7,22]. Moreover, amyloid fibrils exhibit heterogeneity, with the same precursor being able to produce self-propagating fibril polymorphs with distinctive molecular structures, morphologies, and biological properties [26,27].

## 3. The Amyloidoses and Mechanisms of Amyloid Toxicity

Amyloid fibrils generated in amyloidoses represent abnormal aggregates that result from the misfolding of the precursor peptide or protein [6,7]. These amyloid fibrils can deposit into extracellular plaques, as observed in Alzheimer’s disease and dialysis-related amyloidosis, whereas in Parkinson’s and Huntington’s amyloids are present in intracellular inclusions [5]. Identifying the species produced in amyloid assembly reactions that are the culprits of toxicity in amyloidoses and elucidating their mechanisms of action is a priority. A plethora of experimental studies have shown that prefibrillar oligomers produced in the lag phase of amyloid assembly reactions are toxic both in vitro and in vivo [28,29]. Crucially, toxic prefibrillar oligomers are not unique to the peptides and proteins that form amyloids in human disease; they can also be generated from sequences that do not form amyloids naturally [30,31,32]. The implication is that amyloid assembly reactions have the inherent potential to produce toxic species irrespective of the amyloidogenic precursor. Analyses of the mechanisms of toxicity have revealed that cellular membranes are a major target for prefibrillar oligomers, with membrane permeabilisation resulting in elevated intracellular Ca^2+^, the induction of oxidative stress and cell death [31,33,34].

Whilst prefibrillar oligomers are important participants in amyloid disease, they can be transient and heterogenous in nature [28,29]. For example, there are multiple different oligomeric forms of Aβ: dimers, trimers, tetramers, pentamers, decamers, Aβ-derived diffusible ligands, dodecamers, and Aβ*56 [35,36,37,38,39,40]. Moreover, oligomers can exhibit markedly different biological properties, with the same peptide or protein producing both toxic and non-toxic oligomers [41,42]. Adding to this complexity, prefibrillar oligomers are unlikely to be the only toxic species associated with amyloids. Fibrils exhibit a range of disease-relevant properties, including the capacity to damage membranes either via direction interaction or by elongating on the membrane surface [43,44,45,46]. Fibrils can also act as an additional source of oligomers by either shedding from their ends or by providing a surface that catalyses new oligomer formation [45,47,48,49]. Thus, when examining the mechanisms of amyloid toxicity, an array of prefibrillar oligomers, fibrils, and fibril-derived oligomers need to be considered.

## 4. Functional Amyloids and Their Physiological Roles in Humans

Amyloids can also be produced as a natural protein fold with the fibrils performing an array of physiological functions. The fibrous structure of amyloid fibrils facilitates their use as scaffolds for biochemical processes, whereas the compact nature of the amyloid fold is ideal for the storage of proteins and peptides. In humans, several different peptides and proteins have been shown to self-assemble into fibrils that perform physiological functions and that have properties consistent with amyloids [11,12,13,14,15,16,17,18,19,20,21]. The experimental evidence for the formation of amyloid fibrils by these peptides and proteins is summarised in Table 1. For clarity, in this article, the term amyloid is used when a cross-β X-ray fiber diffraction pattern and amyloid-specific dye binding have both been demonstrated. Fibrils that bind amyloid-specific dyes, but that have not been shown experimentally to have a cross-β structure, will be referred to as amyloid-like. 

Amyloid fibrils of pigment cell-specific pre-melanosomal protein (PMEL) are localised to the lumen of melanosomes, a specialised organelle present in the skin and eyes that acts as the site for the synthesis of the pigment melanin [17,50,51]. The fibrils are thought to be scaffolds for the deposition of the tyrosine-based polymer melanin, sequestering toxic intermediates in melanin synthesis [17,50,51]. An analogous scaffold function has been proposed for receptor-interacting protein 1 (RIP1)/RIP3 amyloid fibrils [20]. In regulated necrosis, RIP1 and RIP3 kinases co-assemble into heterodimeric amyloid fibrils that act as a signalling complex known as the necrosome [52]. The necrosome recruits free RIP3, resulting in its autophosphorylation and the subsequent recruitment and phosphorylation of mixed-lineage kinase domain-like (MLKL), a key downstream substrate in regulated necrosis [52].

In cells subjected to stressors amyloid-bodies (A-bodies) act as stores for proteins [11]. Both acidosis and heat shock induce the expression of ribosomal intergenic noncoding RNA (rIGSRNA), which seeds A-body formation in the nucleus [11]. Multiple proteins (>180) assemble into the A-bodies, including proteins involved in cell cycle progression and DNA synthesis, resulting in cells entering a dormant state [11]. Conversely, the removal of the stressor causes A-bodies to disassemble [11]. Similarly, many peptide hormones are stored as amyloid fibrils in the acidic lumen of the secretory granules in endocrine cells [16]. The high density of the amyloid fibrils is thought to give the granules their characteristic dense core. However, upon granule secretion, the fibrils will dissociate into the monomeric form of the peptide hormone due to exposure to the higher extracellular pH [16].

Antimicrobial peptides (AMPs) play a central role in innate immune responses to infection [53]. They share a number of characteristics with the peptides and proteins that form amyloids, with both AMPs and amyloidogenic sequences being able to form pores that permeabilise membranes [53,54]. Moreover, the AMPs LL-37 and protegrin-1 can assemble into amyloid-like fibrils, suggesting that they, and potentially other human AMPs, can also be classified as functional amyloids [15,19].

Functional amyloid fibrils have also been proposed to participate in sexual reproduction [55]. Amyloid fibrils are present in the sperm acrosome, an acidic organelle in the sperm head [12]. These fibrils are thought to facilitate the controlled release of proteins during the acrosome reaction, a key event in the fertilisation of oocytes in which the contents of the acrosome are released [12,55,56]. Amyloids formed by the cystatin-related epididymal spermatogenicis (CRES) subgroup proteins CRES, CRES2, CRES3, and cystatin E2 are present in the epididymis [13,14]. Although the role of amyloids formed by the CRES proteins is not well understood, CRES has antimicrobial activity, is important for the acrosome reaction, and is required for normal lysosomal function in the epididymis [13,57,58,59]. Seminal fluid also contains amyloids and amyloid-like fibrils, formed respectively by peptide fragments of enzyme prostatic acid phosphatase (PAP) and semenogelin proteins (SEM1 and SEM2) [18,21]. Originally identified as enhancers of viral infection [18,21], these fibrils bind to and immobilise damaged sperm cells enabling their clearance by immune cells [60].

Artificial nanomaterials that use amyloid fibrils as a scaffold for the growth of cells can be used as functional amyloids. For example the peptide TTR1-RGD combines residues 105–115 of the human amyloidogenic protein transthyretin and the three amino acid cell adhesion motif RGD from fibronectin [61]. This peptide forms amyloid fibrils that can support the adhesion and growth of fibroblasts [61]. In another study, hen-egg white lysozyme amyloid fibrils were used as a substitute for collagen in a bone biomimetic composite, which was able to support the adhesion and growth of pre-osteoblast cells [62].

## 5. Does Functional Amyloid Fibril Assembly Generate Toxic Species?

Given the potential for the production of toxic species in amyloid assembly reactions, it is remarkable that under normal circumstances cells can produce functional amyloids fibrils without any obvious deleterious consequences. Is this because functional amyloids are intrinsically non-toxic to human cells? A number of studies indicate that this is not the case; thus, the distinction between functional amyloids and pathological amyloids is not always clear-cut.

Amyloid fibrils formed from the peptide hormones glucagon, α-helical corticotropin-releasing factor, glucagon-like peptide 2, and urocortin 3 reduce cell viability when incubated with cultured cells and primary neurons [16]. Likewise, amyloid fibrils formed by the seminal PAP peptide fragment are toxic to a neuronal cell line [63]. Perturbation of PMEL fibril assembly may also result in the production of toxic amyloids. PMEL is synthesised as a transmembrane glycoprotein precursor; subsequent proteolytic processing releases the N-terminal Mα fragment that forms the fibril core [17,50,51,64,65,66,67,68]. The chicken PMEL *Dominant White* (*DW*) mutation results in the formation of abnormal compact fibrillar structures in cells, the loss of melanosomes and reduced cell viability [50,51,69,70]. Crucially, this is a more severe phenotype that of the mouse PMEL knockout, which only results in a modest loss of pigmentation [71]. The *DW* mutant has a three-amino acid insertion in the transmembrane domain, a region of the protein that does not form part of the Mα fibril core [70]. This mutation has no effect on either the trafficking of PMEL to melanosomes or on its proteolytic processing, but instead causes abnormal oligomerisation of the transmembrane domain and/or association with membranes [69]. This may alter the assembly of PMEL resulting in the formation of increased numbers of toxic oligomers or the formation of abnormal fibril polymorphs that are toxic to the cells [69].

Surprisingly, the archetype disease-associated amyloid Aβ may itself be a functional amyloid that acts as an AMP [72,73,74]. Aβ exhibits antimicrobial properties that target pathogenic bacteria and fungi in vitro and in *Caenorhabditis elegans* and mouse models [72,73]. The aggregation of Aβ into amyloids is central to its proposed antimicrobial properties, with assembly intermediates and amyloid fibrils interacting with microbial cell walls to inhibit microbial adhesion to host cells and to agglutinate the microorganisms [72,73,74]. Consistent with an antimicrobial role for Aβ, there are clear parallels with AMPs such as LL-37. Indeed, LL-37 not only exhibits antimicrobial properties and forms amyloid-like fibrils, but can also be toxic to host cells [73,75,76,77,78,79].

The potential for the toxicity of amyloids is also relevant for the biocompatibility of nanomaterials that incorporate amyloid fibrils. Whilst TTR1-RGD amyloid fibrils can support cell growth initially, a reduction in cell viability is observed in longer-term cultures using these fibrils as a scaffold for cells [61,80]. Though in this instance toxicity could be associated with the use of the TTR1 sequence from transythretin, a protein which forms amyloids in transythretin amyloidosis [4,5,6,7].

It is also worth noting that the induction of cell death can be a normal physiological function of amyloids, with RIP1- and RIP3-forming amyloid fibrils in cells undergoing regulated necrosis [20]. Cell death is thought to be induced via the activation of RIP3 kinase substrates and not via the formation of toxic amyloid species [20], although any coincidental toxicity associated with RIP1/RIP3 fibril formation could amplify the necrotic effects of the signaling pathway. RIP1/RIP3 mediated necrosis is also activated in pathological processes such as ischemic injury and damaging inflammatory processes [81]. Thus, even though their normal function is performed, RIP1/RIP3 fibrils can be involved in disease.

## 6. How Do Cells Avert Toxicity in Functional Amyloid Assembly?

**Figure 2:**
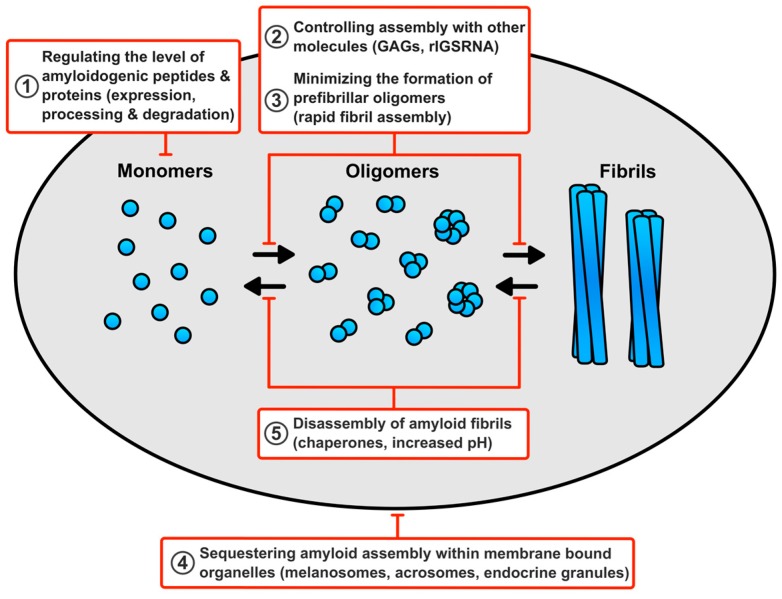
How human cells prevent functional amyloids from causing toxicity? (1) Controlling the level of the amyloidogenic peptides and proteins by regulating its expression, degradation, and generation by proteolysis of a protein precursor will prevent the overproduction of amyloids. (2) The rapid assembly of fibrils will reduce the production of any potentially toxic prefibrillar oligomers. (3) Molecules, such as glycosaminoglycans (GAGs) and ribosomal intergenic noncoding RNA (rIGSRNA), promote amyloid assembly ensuring it only occurs when and where required. These molecules may also promote rapid fibril assembly and prevent unwanted interactions with other cellular components. (4) Sequestering assembly and storage of amyloids within membrane bound organelles will prevent unwanted interactions with other cellular components. (5) The ability to disassemble functional amyloids, under physiological conditions, with chaperones or by exposure to higher pH, will ensure that the fibrils can be removed when no longer required.

The potential for toxicity exhibited by amyloid fibrils and their assembly intermediates highlights that functional amyloid assembly must be tightly controlled. A full picture for how cells prevent functional amyloid toxicity is lacking, but a number of protective mechanisms could come into play (Figure 2).

### 6.1. Regulating the Level of the Amyloidogenic Peptides and Proteins

Elevated levels of amyloidogenic peptides and proteins either through increased expression, decreased degradation or a combination thereof, are thought to be a key factor in the development of amyloidoses [82,83]. For example, Alzheimer’s disease and DRA are associated with elevated levels of monomeric Aβ peptides and β_2_m respectively, which can result in their aggregation [84,85]. Thus, the level of the precursors of functional fibrils amyloids will require tight regulation, as too much of the amyloidogenic precursor could lead to unwanted amyloid formation. Indeed, given the antimicrobial activity of Aβ [72,73,74] and the role attributed to inflammation in Alzheimer’s disease [86], it is tempting to speculate that amyloid formation and cellular toxicity in Alzheimer’s disease could also result from overproduction of Aβ peptides in dysregulated immune responses to either infection or sterile inflammation.

Proteolytic processing may represent one mechanism cells use to regulate the level of amyloidogenic peptides and proteins. Analogous to Aβ peptides, which are generated by cleavage of the amyloid precursor protein by β-secretases and γ-secretases, PMEL is processed by proteases prior to assembling into amyloids [51,64,65,66,67,68,87]. The endoplasmic reticulum is the site of synthesis of PMEL and the protein traffics to by melanosomes via the Golgi apparatus and early endosomes [51]. PMEL is subjected to proteolytic cleavage in the trans-Golgi network and endosomes to liberate the fibril forming Mα fragment [64,65,66,67,68]. Thus, proteolytic processing may not only regulate the level of the Mα fragment, but it will also ensure that PMEL amyloid fibrils do not assemble prematurely in the secretory pathway. Proteolytic processing may also regulate the production of seminal amyloid and amyloid-like fibrils, which are formed by peptide fragments of PAP and semenogelin proteins [18,21].

### 6.2. Minimising the Production of Prefibrillar Oligomers

Little is known about the oligomers produced in the assembly of functional amyloids. Nonetheless, these assembly reactions must pose a particular risk for cells due to the inherent potential for the production of toxic oligomers when amyloids are assembled [30,31,32]. Indeed, both acrosomes and the epididymis contain material recognised by the antibody A11, which binds to toxic prefibrillar oligomers generated from an array of different amyloidogenic precursors [12,13,25]. Thus, it may not be possible to prevent the formation of toxic prefibrillar oligomers in functional amyloid assembly. Functional amyloid assembly may instead minimise the formation of toxic prefibrillar oligomers to below a threshold that causes deleterious effects. PMEL may achieve this by assembling into fibrils at a rate that is orders of magnitude faster than that observed for the disease-associated sequences Aβ_1-40_ and α-synuclein [17]. This rapid assembly of fibrils should minimise the level of prefibrillar PMEL oligomers, limiting their ability to cause toxicity. It is unknown, however, whether rapid assembly is a feature of other functional amyloid fibrils. Furthermore, it is important to note that prefibrillar oligomers are only one potential source of toxicity, with mature amyloid fibrils and fibril-derived oligomers also exhibiting deleterious effects [43,44,45,47,48,49].

### 6.3. Controlling Assembly of Functional Amyloid Fibrils with other Molecules

An additional level of regulation to the assembly of a number of functional amyloid fibrils may be provided by molecules that promote fibril assembly, ensuring fibrils only form when and where required. Proteins stored in A-bodies have an amyloid-converting motif (ACM), which corresponds to an arginine/histidine rich region flanking an amyloidogenic stretch of residues [11]. Yet these proteins do not form amyloids spontaneously in cells. Instead expression of rIGSRNA induced by stressors is required to seed assembly of proteins with ACMs into fibrils, potentially via interaction between the RNA and the arginine/histidine rich region flanking the amyloidogenic stretch [11]. Similarly, many of the peptide hormones stored within endocrine granules as amyloid fibrils do not form fibrils spontaneously in vitro at the acidic pH of the granule lumen [16]. Amyloid formation by many of these peptides is stimulated by glycosaminoglycans (GAGs), which are also present within endocrine granules [16,88]. In addition to their role in promoting functional amyloid assembly these molecules may help to prevent toxicity. Indeed, it has been shown that GAGs by accelerating fibril formation can reduce toxicity associated with amyloid assembly reactions, presumably by lowering the level and lifespan of prefibrillar oligomers [89,90,91]. Moreover, GAGs can prevent the disruption of lipid membranes by preformed amyloid fibrils [92].

### 6.4. Sequestering Functional Amyloid Assembly Reactions within Membrane Bound Compartments

PMEL assembles into fibrils within early endosomes and melanosomes, whereas amyloid fibrils formed by peptide hormones and acrosomal matrix proteins assemble within the lumen of endocrine granules and acrosomes, respectively [12,16,17]. These membrane bound organelles provide an acidic environment that is optimal for the assembly of the amyloid fibrils [12,16,17,50,51]. The surrounding membrane may also act as a physical barrier to prevent unwanted interactions between amyloids and other cellular components. Yet, the sequestration of amyloids within membrane bound organelles may itself present a problem. Membranes are a key cellular target in amyloidoses, with prefibrillar oligomers, amyloid fibrils, and fibril-derived oligomers being shown to permeabilise lipid membranes [33,34,43,44,45]. The extent of membrane disruption is, however, dependent on the lipid composition of the membranes [93,94]. This may be reflected in the lipid profiles of organelles in which functional amyloids are assembled.

### 6.5. Disassembly of Functional Amyloid Fibrils

Amyloidoses are characterised by the progressive accumulation of amyloid deposits, suggesting that these diseases are caused, at least in part, by a failure to remove these unwanted protein aggregates [82,83]. In contrast, a number of functional amyloids have been shown to disassemble readily under physiological conditions. A-body formation in the nucleus is reversible, the removal of the stressors resulting in their chaperone-dependent disassembly [11]. PMEL amyloid fibrils, as well as those formed by peptide hormones and acrosomal proteins are localised to acidic organelles and disassemble when exposed to neutral pH [12,16,95,96]. Moreover, no stable oligomers are observed when fibrils of the PMEL repeat domain, which is part of the Mα fragment, disassemble at neutral pH [96]. The ability to disassemble rapidly into monomers is clearly important for the function of peptide hormones and may facilitate the dispersal of acrosomal matrix proteins during the acrosome reaction. It will also ensure that fibrils released by cells disassemble into monomers before either the fibrils or fibril-derived oligomers have the opportunity to cause any deleterious effects. Similarly, if the membranes surrounding the organelles in which amyloid fibrils are localised become damaged, any fibrils that inadvertently access the cytosol would presumably also rapidly disassemble.

## 7. Summary and Remaining Questions

In summary, functional amyloid fibrils participate in an array of physiological processes, but their assembly could pose significant risks for cells. A number of mechanisms that may help avert the toxicity of functional amyloids and their assembly intermediates can be proposed. These include regulating the levels of amyloidogenic peptides and proteins, minimising the formation of prefibrillar oligomers, sequestering fibril assembly inside membrane bound compartments, regulating assembly with GAGs and RNAs and being able to disassemble amyloid fibrils. However, questions still remain about why functional amyloids have no pronounced ill effect on the cells responsible for their production.

- Does the dysregulation of functional amyloid production result in disease? Of particular interest is whether Alzheimer’s disease is caused by the overproduction of a functional amyloid.
- Do functional amyloid assembly reactions produce toxic oligomers? Studies suggest that toxic prefibrillar oligomers are a common feature of amyloid assembly, yet surprisingly little is known about the properties of oligomers associated with functional amyloids.
- Are functional amyloids able to assemble more rapidly than disease-associated amyloids, thus limiting the production of any toxic prefibrillar oligomers?
- In addition to promoting the assembly of functional amyloids do rIGSRNA and GAGs also prevent amyloid toxicity?
- How can functional amyloid fibrils be assembled and stored within membrane bound compartments when cellular membranes represent a key target in amyloid toxicity?

Crucially, a better understanding of how cells prevent toxicity in the production of functional amyloids will provide insights into how to prevent tissue degeneration in amyloidoses.

## Figures and Tables

**Table 1:** Functional amyloids: summary of the experimental evidence for the formation of amyloid fibrils.

Protein, Peptide or Cellular Structure	Proposed Functions	Experimental Evidence for Amyloid Fibrils
Amyloid-bodies (A-bodies)	Stores of proteins in stressed cells	A-bodies are stained by Congo red and thioflavin-S. Proteins that accumulate in A-bodies can form fibrils with a cross-β X-ray fiber diffraction pattern [11].
Acrosomes	The acrosome reaction during fertilisation of oocytes.	Acrosomes in sperm are stained by thioflavin-S and are recognised by amyloid-specific antibodies. Purified acrosomal matrix has a cross-β X-ray fiber diffraction pattern [12].
Cystatin-related epididymal spermatogenicis (CRES) subgroup proteins	Antimicrobial activity, acrosome reaction and lysosomal function	Material from the epididymis has a cross cross-β X-ray fiber diffraction pattern, is recognised by amyloid-specific antibodies and binds thioflavin-S and Congo red. CRES proteins co-localises with thioflavin-S. Fibrils of CRES proteins bind thioflavin-T and are recognised by amyloid-specific antibodies [13,14].
LL-37	Antimicrobial	Fibrils exhibit green birefringence with Congo red [15].
Peptide hormones	Storage of the hormone in secretory granules	Purified granules from endocrine cells have a cross-β X-ray fiber diffraction pattern and exhibit green birefringence with Congo red. The fibrils bind Congo red and have a cross-β X-ray fiber diffraction pattern [16].
Pigment cell-specific pre-melanosomal protein (PMEL)	Pigmentation	Purified melansomes are stained by thioflavin-S and Congo red. The fibrils have a cross-β X-ray fiber diffraction pattern, bind Congo red and thioflavin-T and have a far ultraviolet circular dichroism spectrum consistent with β-sheet content [17].
Prostatic acid phosphatase peptides	Removal of damaged sperm	The fibrils have a cross-β X-ray fiber diffraction pattern, bind thioflavin-T and exhibit green birefringence with Congo red [18].
Protegrin-1	Antimicrobial	The fibrils bind thioflavin-T [19].
Receptor-interacting protein 1 (RIP1)/RIP3	Regulated necrosis	The fibrils have a cross-β X-ray fiber diffraction pattern, a solid state NMR spectra consistent with a β-sheet core and bind thioflavin T and Congo red [20].
Semenogelin proteins (SEM1 and SEM2)	Removal of damaged sperm	The fibrils bind thioflavin-T and Congo red and an amyloid-specific antibody pulls down SEM 1 and SEM 2 from seminal fluid [21].
